# Correlation Study of Antioxidant Activity with Phenolic and Flavonoid Compounds in 12 Indonesian Indigenous Herbs

**DOI:** 10.3390/antiox10101530

**Published:** 2021-09-27

**Authors:** Yeni Maulidah Muflihah, Ganesh Gollavelli, Yong-Chien Ling

**Affiliations:** 1 Department of Chemistry, National Tsing Hua University, Hsinchu 30013, Taiwan; yeni.maulidah.fmipa@unej.ac.id; 2 Department of Chemistry, Faculty of Mathematics and Natural Sciences, University of Jember, Jember 68132, Indonesia; 3 Department of Humanities and Basic Sciences, Faculty of Engineering Chemistry, Aditya Engineering College, Surampalem, Jawaharlal Nehru Technological University, Kakinada 533437, India; ganeshg@aec.edu.in

**Keywords:** antioxidant activity, correlation analysis, flavonoid, Indonesian herbs, phenolic, mixed-method

## Abstract

The antioxidant activity (AA), total phenolic content (TPC), and total flavonoid content (TFC) of selected Indonesian *Zingiberaceae* herbs were determined. An optimization extraction procedure was conducted by using Taguchi L_16_ orthogonal array. Four chemical assays were applied, including 2,2-diphenyl-2-picrylhydrazyl (DPPH) radical scavenging activity assay, H_2_O_2_ scavenging activity assay, Folin–Ciocalteau (F–C) assay, and NaNO_2_-AlCl_3_-NaOH assay, which revealed remarkable differences in AA, TPC, and TFC. The result indicated the diversity of AA composition among the herbs, and *C. longa* exhibited the highest AA. HPLC-PAD analysis revealed that curcumin was present in five high antioxidant herbs, and the highest amount was in *C. longa*. Pearson correlation analysis indicated that the identified TPC and TFC were significant contributors to AA, and curcumin was likely the main contributing antioxidant compound. Our approach concluded that *C. longa* is the greatest source of natural antioxidants among 12 Indonesian indigenous *Zingiberaceae* herbs. The use of a mixed-method approach to augment the findings of solitary methods might facilitate future researchers to uncover deeper and hidden meanings.

## 1. Introduction

The antioxidants are generally known as natural or synthetic compounds which avoid or prolong the damage of cells in the presence of reactive oxygen by opposing the oxidation process or inhibiting the reaction promoted by oxide or peroxide species, mostly known as free radical oxygen species [1]. The free radicals generated during cell metabolism will be deactivated and stabilized by the antioxidants before and after attacking the targets in the biological system [2]. Effective antioxidants break down the radical chain reaction and act as radical scavengers [1,3]. Antioxidants therefore play a predominant role as stabilizers to maintain biological functions without fail.

Antioxidants are from either natural or synthetic source. Natural plant extracts are usually rich in antioxidants, which are good sources for food additives, medicine, and cosmetics purposes [4]. In food, antioxidants are needed to prevent food deterioration during storage or processing and maintain the food quality of freshness, nutrients, texture, aroma, and functionality [5]. Antioxidants are present in food itself or by external addition. In medicinal use, antioxidant activity is generally due to their ability to exhibit radical scavenging capacities. The phytochemicals such as phenolics, flavonoids, anthocyanins, carotenoids, ascorbic acids, terpenoids, tannins, and tocopherols in medicinal plants are known for preventing and curing disease [6]. In cosmetics, the effective use of topical antioxidants to improve the protection system of endogenous cutaneous is well known [7]. Synthetic antioxidants are ineffective in some formulations because of their instability and low absorption in the aqueous media. Furthermore, some active ingredients in synthetic antioxidants might exhibit contradictory reactions leading to problems such as allergies, irritant contact dermatitis, phototoxicity, and photo allergies [7,8]. Natural plant antioxidants and their derivatives used in anti-aging cosmetics exhibit better performance and less toxicity over synthetic antioxidants and are gaining interest worldwide [2,9].

Indonesia, located in Southeast Asia, inherits rich biodiversity and traditional medicine. Around 2500–7500 plant species out of 30,000–40,000 plant species are medicinal plants [10]. The Indonesian indigenous *Zingiberaceae* herb family is the most widely applied and known for its potency as a natural antioxidant. These plants contain mainly phenolics, terpenoids, and alkaloids [11,12]. Studies investigating *Zingiberaceae* as prospective antioxidant sources have increased significantly. The antioxidant activity (AA) property of *Zingiberaceae* plants is related to their chemical composition primarily attributed to their richness in total phenolic content (TPC), total flavonoid content (TFC), terpenoids, alkaloids, and organic acids, with curcumin as the main antioxidant constituent in most of the plants from this family [3,11,13,14]. Ultrasonic extraction [15] is one of the prominent extraction techniques along with supercritical fluid extraction [16], microwave-assisted extraction [17], and hot water extraction [18]. 

The purpose of this study was to determine the AA as well as the TPC and TFC by chemical assays in 12 Indonesian indigenous *Zingiberaceae* herbs, to find promising natural antioxidants for future use in diverse fields such as food, medicine, and cosmetics. It was desirable to select the specific assay based on fitness for purpose. For example, cell lines testing might be needed for cosmetic purpose. Herein, we used a mixed-method approach [19] integrating both qualitative and quantitative methods for sample collection, chemical analysis, correlation analysis, and result interpretation. Optimum ultrasonic extraction conditions efficiently obtained via Taguchi experiment design [20] was adopted for antioxidant extraction first. The preliminary chemical assay results were further confirmed by high-performance liquid chromatograph−photodiode array detector (HPLC-PAD) instrumental analysis and correlation analysis to find out the major active compounds and components causative to the AA.

## 2. Materials and Methods

### 2.1. Chemicals and Reagents

All chemicals of either LC grade or analytical grade have been purchased from various suppliers. In brief, 2,2-diphenyl-2-picrylhydrazyl (DPPH, ≥99%), Folin–Ciocalteau reagent, formic acid (ACS reagent purist. p.a.), aluminum chloride anhydrous, ethanol (LC grade, 99%), sodium phosphate dibasic (99%), hydrogen peroxide (technical, 30% (*w*/*w*), in water), and sodium phosphate monobasic (99%), were purchased from Sigma Aldrich (St. Louis, MO, USA); gallic acid monohydrate (ACS reagent, 98%), naringin, curcumin (≥99%), and quercetin (≥95% HPLC, solid) were from Fluka (Buchs, Switzerland); ascorbic acid and methanol were from Tedia (Fairfield, OH, USA). Ferulic acid was from TCI (Tokyo, Japan), chlorogenic acid was from Acros organic (Schwerte, Germany); sodium hydroxide was from Macron Fine Chemicals (Lehigh, PA, USA); sodium carbonate anhydrous was from Merck (Darmstadt, Germany); and sodium nitrite from Showa (Tokyo, Japan). 

### 2.2. Herb Sample Collection and Preparation

**Figure 1:**
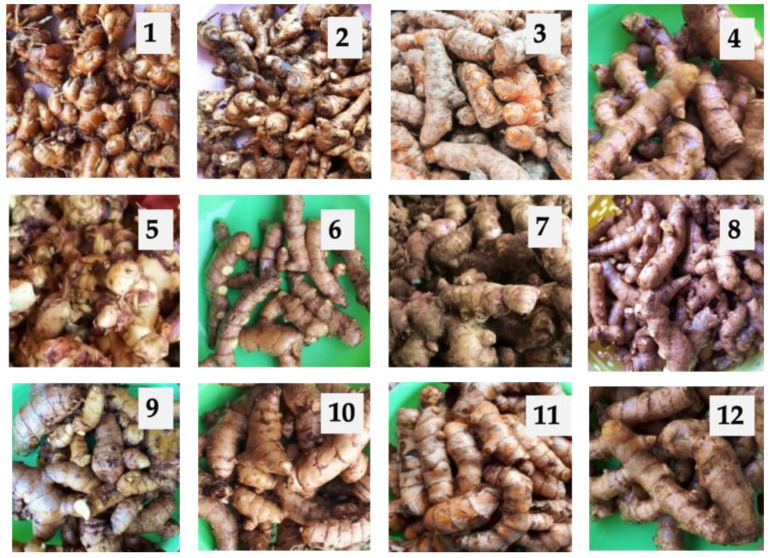
The rhizome part of 12 Indonesian indigenous *Zingiberaceae* species herbs: (**1**) *K. galanga*, (**2**) *B. rotunda*, (**3**) *C. longa*, (**4**) *Z. cassoumounar*, (**5**) *C. heynana*, (**6**) *A. purpurata*, (**7**) *Z. officinale*, (**8**) *Z. zerumbet*, (**9**) *C. manga*, (**10**) *C. aeruginosa*, (**11**) *C. xanthorrhiza*, and (**12**) *C. zedoaria*.

The rhizome part of *Zingiberaceae* species of 12 Indonesian indigenous herbs traditionally used in cosmetics and medicines was used. The species included *Zingiber officinale* (*Z. officinale*), *Zingiber zerumbet* (*Z. zerumbet*), *Zingiber cassumunar* (*Z. cassumunar*), *Curcuma aeruginosa* (*C. aeruginosa*), *Curcuma xanthorrhiza* (*C. xanthorrhiza*), *Curcuma longa* (*C. longa*), *Curcuma manga* (*C. manga*), *Curcuma heynana* (*C. heynana*), *Curcuma zedoaria* (*C. zedoaria*), *Alpinia purpurata* (*A. purpurata*), *Boesenbergia rotunda* (*B. rotunda*), and *Kaempferia galangal* (*K. galangal*) (Figure 1), and all were purchased in August 2018 at a traditional market in Jember, East Java, Indonesia. Before extraction, the samples were cleaned, chopped, dried for two days, grinded into powder, and stored in vacuum plastic bags. The water content of the samples was determined by using an oven at 105 °C in triplicate.

### 2.3. Taguchi Experimental Design for Ultrasonic Extraction

A popular Taguchi L_16_ orthogonal array experimental design was adopted to find the optimum ultrasonic extraction [15]. This design involved three variable factors (ethanol concentration, extraction time, and solid-to-liquid ratio), each at four levels, with a total of 16 experimental measurements using *C. zedoaria* as a representative sample. Three fixed factors were temperature (32 °C), frequency of the ultrasonic bath (40 Hz), and volume of ethanol added (10 mL). Our primary goal was to identify herbs with high AA by a mixed-method approach; we therefore focused on AA optimization first. The larger-the-better signal-to-noise ratio (SN ratio) for AA was selected to find the optimum extraction conditions, and described as follows:

(1) $$\frac{S}{N}=-10\;\log\left(\frac{1}{n}\sum_{i=1}^{i=n}\frac{1}{{y_{i}}^{2}}\right)$$

where **y_i_** denotes the **i**th measured AA, and ***n*** is the total number of measurements.

The appropriate amounts of powdered samples were weighed and soaked with different concentrations (50%, 60%, 70%, and 80%) of 10 mL ethanol in a tube and ultrasonically extracted in an ultrasonic bath Delta DC150H (Delta Ultrasonic CO., Ltd., Taiwan). The obtained extracts were filtered by using Whatman™ no. 42 filter paper and dried using N_2_ fluxes. The dried extract was then weighed (approximately 2–5 mg); an aliquot of 10 mL 99% methanol was added, vortexed, and filtered by using a PTFE 0.22 μm syringe filter. The herb extract stock solutions were stored in glass containers at 4 °C prior to analysis.

### 2.4. Determination of Antioxidant Activity

The antioxidant activity (AA) was determined by using both DPPH free radical and H_2_O_2_ scavenging activity assays. In the following, AA represented the measured AA by DPPH free radical scavenging assay result, whereas AA (H_2_O_2_) represented the H_2_O_2_ scavenging activity assay result as a comparison use.

In the DPPH free radical scavenging assay, prior to AA determination, 0.2 mL herb extract stock solution was diluted with 0.2 mL of 15% methanol to yield 10 mg/mL herb extract solution. The 0.5 mg/L test solution was prepared by adding 0.20 mL herb extract solution to 3.80 mL DPPH solution. The solution was shaken vigorously and allowed to stand for 30 min at room temperature in the dark. The DPPH solution and ascorbic acid were used as the control and standard, respectively. Daily prepared standard calibration curves of ascorbic acids (40 to 250 µg/mL) were used to calculate the AA. The absorbance was measured at 515 nm in a T60 UV-Visible spectrophotometer.

Similarly, in the H_2_O_2_ scavenging activity assay, a 40 mM H_2_O_2_ solution was prepared before the AA (H_2_O_2_) determination by dissolving 0.453 mL of 30% H_2_O_2_ in 100 mL of 0.1 M phosphate buffer (pH 7.4). A 0.5 mL herbs extract stock solution was diluted with 0.5 mL methanol to obtain a concentration of 10 mg/mL herbs extract solution. The test solution was prepared by adding 1.0 mL of herb extract solutions to a 0.6 mL of 40 mM H_2_O_2_ solution and 3.40 mL phosphate buffer (pH 7.4). The solution was shaken vigorously and allowed to stand for 10 min at room temperature in the dark before measurement. The H_2_O_2_ solution and ascorbic acid were respectively used as the blank control and standard. Daily prepared standard calibration curves of 50 to 800 µg/mL ascorbic acid were used to calculate the AA (H_2_O_2_). The absorbance was measured at 230 nm in a T60 UV-Visible spectrophotometer. 

The antioxidant activity of both DPPH free radical and H_2_O_2_ scavenging activity assays can be calculated as follows:

(2) $$\mathrm{Antioxidant}\;\mathrm{activity}\;\left(\%\right)=\left[\frac{\left(A_{0}-A\right)}{\left(A_{0}\right)}\right]\times 100\%$$

where **A_0_** is the blank control absorbance and **A** is the sample absorbance.

### 2.5. Determination of TPC

The total phenolic content (TPC) was determined by using the Folin–Ciocalteau (F–C) assay described by Li, et al. [3], but with minor modifications. An aliquot of 400 µL extract was mixed with 2.0 mL of 10% F–C reagent and 1.60 mL 7.5% Na_2_CO_3_ solution. The mixture solution was shaken for 5 min and allowed to stand for 15 min at 37 °C followed by incubation in the dark for 1 h. The same T60 UV-Visible spectrophotometer was used to measure the absorbance at 725 nm to determine the TPC. Daily prepared standard calibration curves of gallic acid in methanol (20 to 250 µg/mL) was used to calculate the TPC, expressed as milligrams of gallic acid equivalents per gram of dried powdered sample (mg GAE/g). 

### 2.6. Determination of TFC 

The total flavonoid content (TFC) was determined by using the NaNO_2_-AlCl_3_-NaOH assay by Li et al. [3], but with minor modification. An aliquot of 0.40 mL extract (or standard) was mixed with 0.3 mL 5% NaNO_2_ and 2.0 mL distilled water. The mixture solution was allowed to stand for 5 min and followed by the addition of 0.3 mL 10% AlCl_3_ and stood for another 6 min. After 1 min, the mixture solution was mixed with 2.0 mL 1.0 M NaOH and 3.2 mL distilled water, mixed with a vortex and stood for 15 min at room temperature. The same T60 UV-Visible spectrophotometer was used to measure the absorbance at 422 nm to determine the TFC concentration. Daily prepared standard calibration curves of quercetin (20 to 600 µg/mL) were used to calculate the TFC, expressed as milligrams of quercetin equivalents per gram of dried powdered sample (mg QE/g).

### 2.7. Determination of SPC Content

The selected phenolic compounds (SPC) content was separated and identified on HPLC-PAD system consisting of a Shimadzu Nexera-i LC-2040C LC, a LC-2040 autosampler, a LC 2040 column oven, and a LC-2030/2040 PDA. An aliquot of 10 μL extract was injected into the HPLC followed by separation performed at a flow rate of 1.0 mL/min on an Agilent Zorbax Eclipse Plus C_18_ column (5 mm particle size, 150 mm length × 2.1 mm ID). Mobile phase A was 1.0% formic acid in deionized water and mobile phase B was methanol. The initial mobile phase was 5% B and increased to 90% B in 20 min gradient elution time. The column oven temperature is accordingly increased from initial 25 °C to final 90 °C. The absorbance was scanned in a range from 190 to 800 nm. Five SPCs, namely curcumin, ferulic acid, naringin, gallic acid, and quercetin were used daily to prepare the standard calibration curves. Other eluting peaks were tentatively identified by comparing their retention time to the respective literature reported retention time. 

### 2.8. Statistical Analysis

All experiments were performed in triplicate, unless otherwise stated. The results were presented as mean ± SD (standard deviation). Taguchi experimental design results were analyzed by using Minitab 19 from Minitab, LLC (State College, PA, USA). The correlation analysis results of AA with TPC, TFC, and SPC were expressed as Pearson correlation coefficients using SPSS Version 24.0 (SPSS, Chicago, IL, USA). The slope of the calibration curve and the coefficient of determination (R^2^) were obtained by using MS Excel 2010 from Microsoft (Redmond, WA, USA).

## 3. Results

### 3.1. Optimum Conditions for Ultrasonic Extraction

An orthogonal array L_16_ design was implemented to the ultrasonic extraction. Solvent concentration, extraction time, and solid-to-liquid ratio (S-L) were selected parameters that could affect the antioxidant compounds extracted [21]. Using *C. zedoaria*, one of Indonesian indigenous herbs that is already known as a traditional medicine, with high AA as a representative sample, we see that the measured TPC, TFC, and AA values differ to a large extent with varying extraction parameters (Table 1).

**Figure 2:**
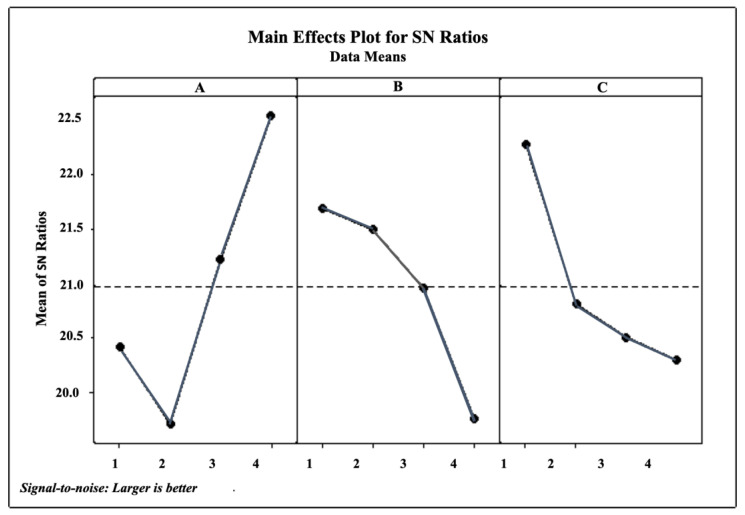
Main effects plot for SN ratio of AA. (**A**) Solvent concentration (50, 60, 70 and 80%), (**B**) extraction time (50, 60, 70 and 80 min), and (**C**) solid-to-liquid ratio (0.02, 0.04, 0.06 and 0.08 g/mL).

The overall optimum conditions selected were based on the main effects plot of SN ratio. Herein, we selected the larger-the-better SN ratio approach to find the optimum conditions for AA (Figure 2). 

Ethanol concentration exhibited positive effect on higher AA (Figure 2A) was also obtained from previous AA compounds (phenolics, flavonoids, tannin, alkaloids) isolated from various samples such as the extraction of 93 Chinese medicines [3], *Zingiberaceae* species [11,20], *Ipomoea batata* leaves [12], and Algerian medicinal plants [22].

The calculated SN ratio of the AA response (Table 2) shows that the influence of solvent concentration has the main effect on the antioxidant activity of the sample, followed by the solid-to-liquid ratio (S–L) and the extraction time.

### 3.2. TPC, TFC, and Antioxidant Activity

**Figure 3:**
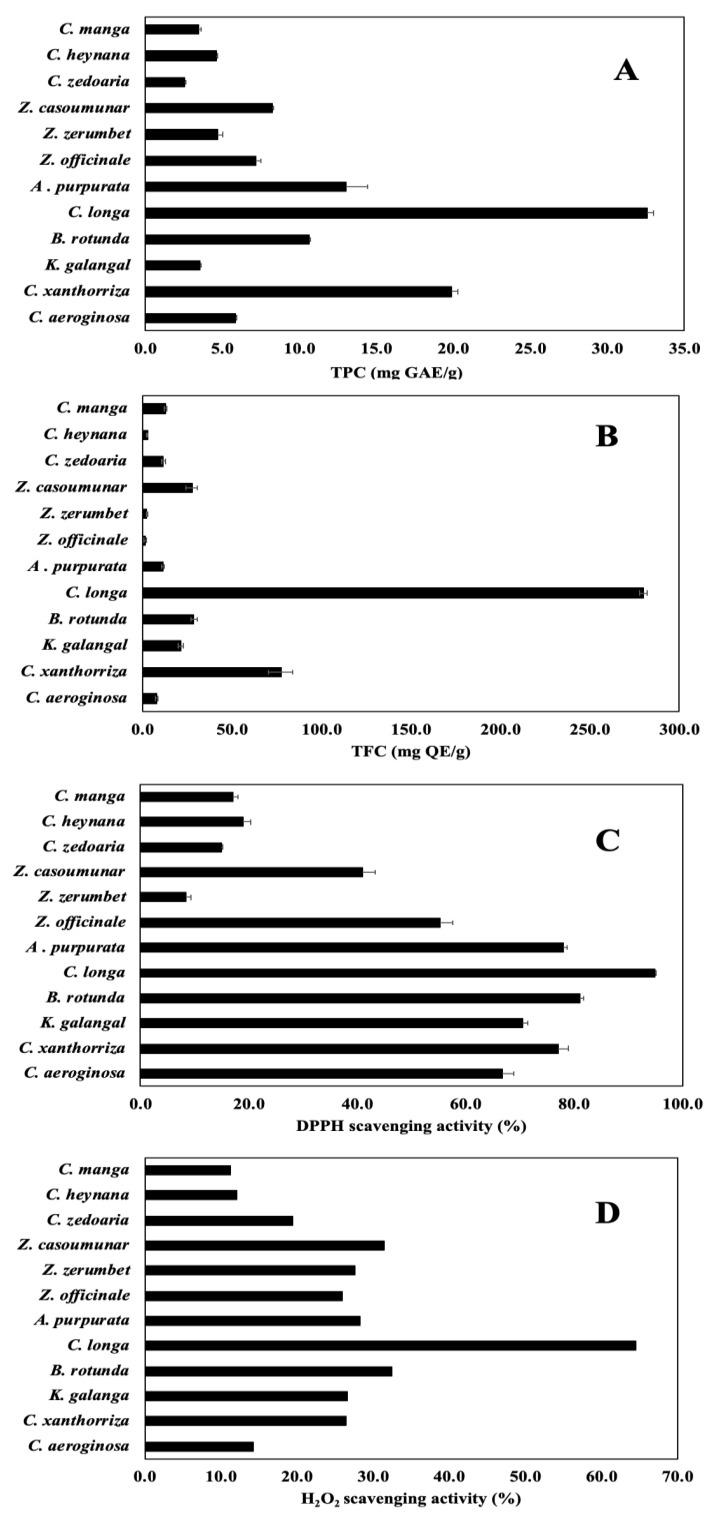
Chemical content and AA in Indonesian herbs: (**A**) TPC by Folin–Ciocalteau assay, (**B**) TFC by NaNO_2_-AlCl_3_-NaOH assay, (**C**) AA, and (**D**) AA (H_2_O_2_), in 12 Indonesian herbs. (*n* = 3, error bars represent standard deviation).

TPC and TFC have been considered major contributors to plant AA [3]. The TPC (as mg GAE/g) was 32.57, i.e., the highest, in *C. longa* and was 2.55, i.e., the lowest, in *Z. zerumbet* (Figure 3A). The TFC (as mg QE/g) was 279.87, i.e., the highest, in *C. longa* and was 1.31, i.e., the lowest, in *Z. officinale* (Figure 3B).

The plant AA was caused by the presence of different antioxidant components in plant tissue. It was measured based on the well-known DPPH assay [23] and H_2_O_2_ assay. The AA (as %) was found to be 94.85, i.e., the highest, in *C. longa* and 8.45, i.e., the lowest, in *Z. zerumbet* (Figure 3C), while the AA (H_2_O_2_) (as %) was 64.46, i.e., the highest, in *C. longa* and 11. 27, i.e., the lowest, in *C. manga* (Figure 3D).

Among the 12 Indonesian herbs investigated, the *C. longa*, *C. xanthorrhiza*, *A. purpurata*, *B. rotunda*, and *K. galangal* presented the highest AA, TPC and TFC obtained.

### 3.3. Correlation Analysis of AA with TPC and TFC

**Figure 4:**
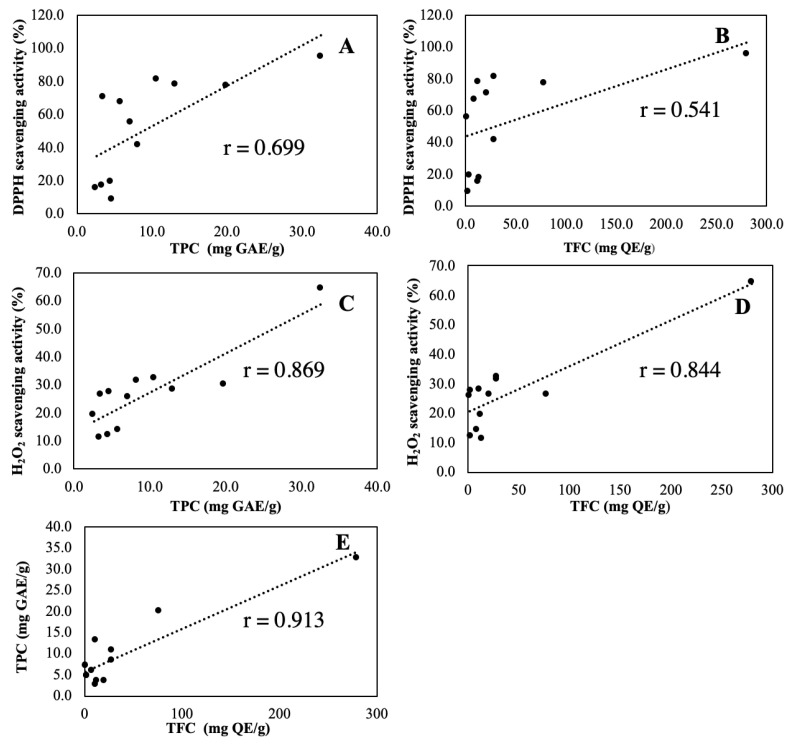
Pearson correlation scatter plot of relationship between (**A**) AA and TPC, (**B**) AA and TFC, (**C**) AA (H_2_O_2_) and TPC (**D**) AA (H_2_O_2_) and TFC, and (**E**) TPC and TFC.

Typical TPC contributing more to plant AA were mainly phenolic acids and flavonoids. We adopted the Pearson correlation coefficient (PCC), also referred to as Pearson’s *r*, to express the strength and direction of the linear relationship of correlation. The PCC scatter plots of AA, TPC and TFC are shown in Figure 4.

### 3.4. Correlation Analysis of AA with SPC

**Figure 5:**
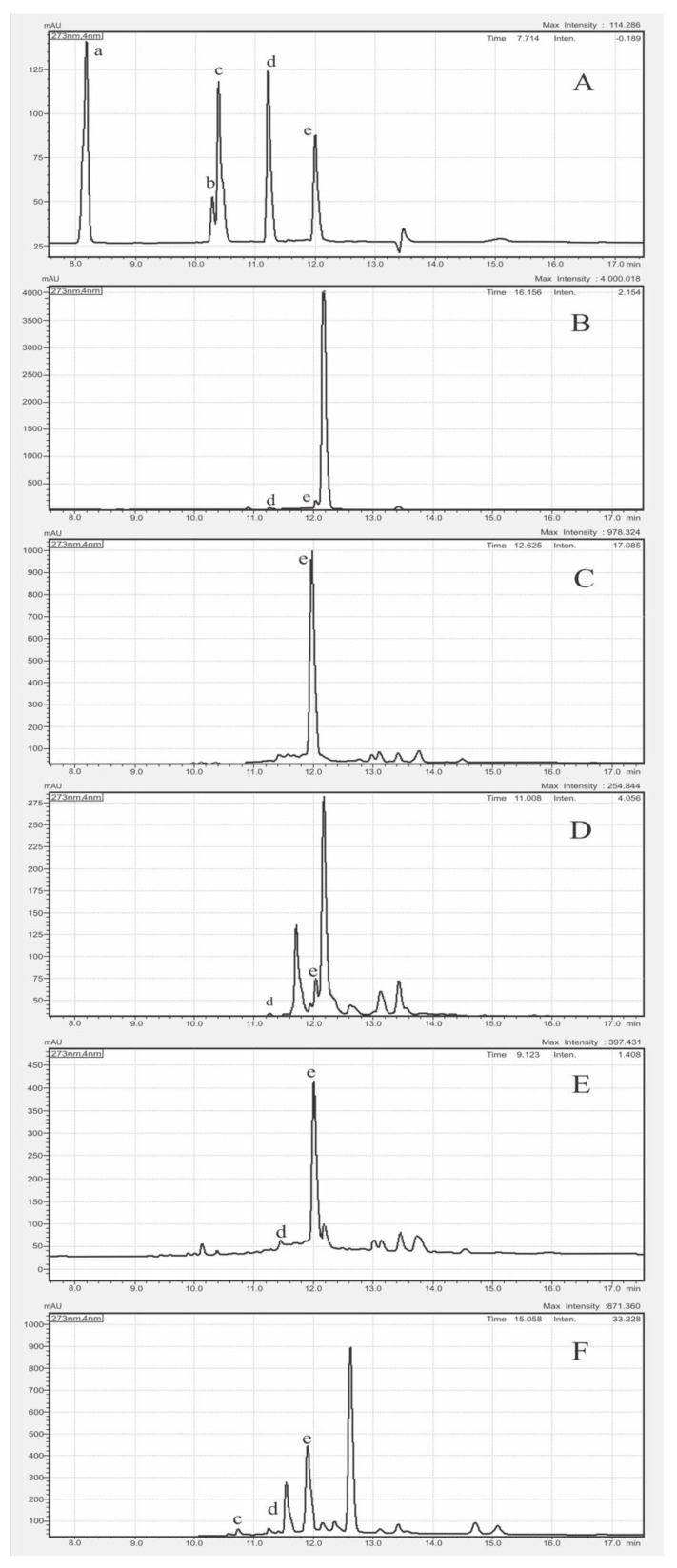
HPLC-PDA chromatograms of (**A**) five SPC standard solutions, (**B**) *K. galangal*, (**C**) *C. longa*, (**D**) *A. purpurata*, (**E**) *C. xanthorrhiza.*, and (**F**) *B. rotunda*. (a: gallic acid, b: naringin, c: ferulic acid, d: quercetin, e: curcumin).

The *C. longa*, *B. rotunda*, *A. purpurata*, *C. xanthorrhiza*, and *K. galanga* exhibiting high TPC, TFC, and AA were further confirmed and quantified for five SPC. The analytical figures of merit (Table 3) of the adopted HPLC-PAD method fit our research purpose. The chromatograms of the five SPC standards and five highest AA herbs are shown in Figure 5.

The SPC content measured by HPLC-PAD (Table 4) revealed that curcumin and quercetin were the most detected SPCs.

**Figure 6:**
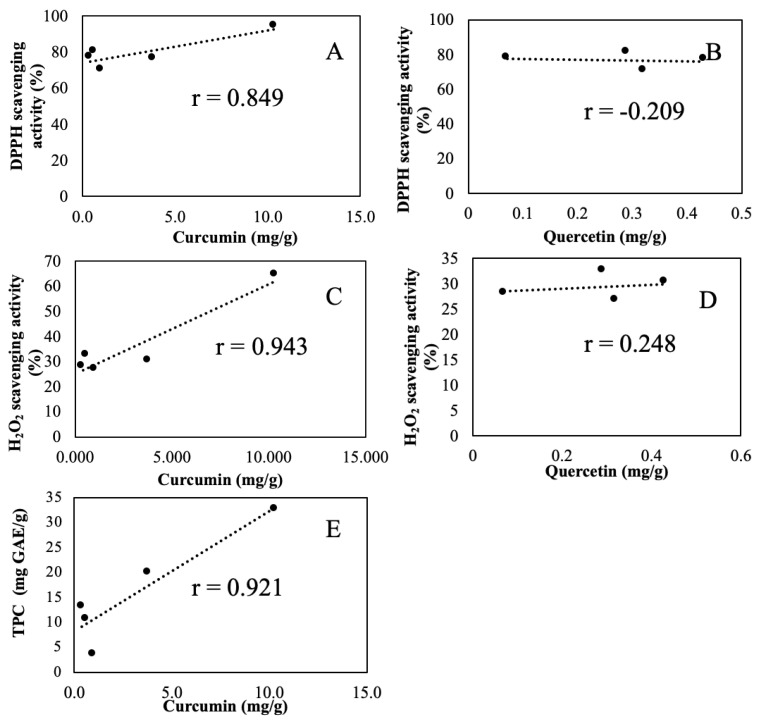
Pearson correlation scatter plots of relationship between (**A**) AA and curcumin, (**B**) AA and quercetin, (**C**) AA (H_2_O_2_) and curcumin (**D**) AA (H_2_O_2_) and quercetin, and (**E**)TPC and curcumin.

The correlation relationships between AA and curcumin/quercetin as well as between TPC and curcumin are shown in Figure 6.

## 4. Discussion

The adoption of L_16_ orthogonal array design in ultrasonic extraction of Indonesian selected herb was a realistic alternative to reduce the experimental trials while achieving similar optimum conditions [15]. We selected the less reported *C. zedoaria* as a representative sample for initial optimization, as we were also interested in studying the matrix effect. *C. zedoaria* might contain AA compounds different from *C. longa*, the most common herbs in *Zingiberaceae* family. Table 1 that shows the measured TPC, TFC, and AA values differ to a large extent with varying extraction parameters. In a set of 16 experiments, the corresponding change ranged from 1.51 to 8.25 mg GAE/g, 2.59 to 25.27 mg QE/g, and 7.97 to 15.94%, respectively. The results indicated that under respective optimum conditions the enhancement factors (the ratio of the highest to the lowest measured values) were TPC~6, TFC~10, and AA~2. The increasing AA activity of *C. zedoaria* indicated that the number of extracted AA compounds was highly affected by the extraction conditions which were dictated by the characteristics of the targeted antioxidant compounds. This observation also holds true for known TPC and TFC compounds. Figure 2 shows that the overall optimum conditions selected, based on the main effects plot of SN ratios of AA was 80% ethanol concentration, 50 min extraction time, and 0.02 S-L. The ethanol concentration exhibited a positive effect on higher AA (Figure 2A). This result was reached presumably because at higher concentration; the ability of ethanol to degrade the nonpolar cell walls via semi-polar interactions was enhanced, leading to the increasing release of intracellular phenolic and flavonoid compounds. Ethanol was among the best extraction solvents irrespective of the extraction method [3,11,13,15,17], and increasing ethanol concentration generally led to higher TPC yield [13,15,17]. Longer extraction time exhibited a negative effect of lower AA (Figure 2B), which was presumably due to the resultant prolonged heat exposure leading to the decreasing amount of targeted AA compounds [21,24]. The larger S–L also exhibited a similarly negative effect of lower AA (Figure 2C), presumably due to the smaller solvent volume available to penetrate the sample and leading to reduced solubilizing of the AA compounds. 

The calculated SN ratio for each factor (Figure 2) is presented numerically in Table 2. The table contains a column for each factor (solvent %, time min, and S–L g/L), and each table contains six rows for SN ratio at each factor level followed by delta and rank. Delta is the difference between the maximum and minimum average SN ratio for the factor. The factor with the largest delta ranks as the first, which means the highest influencing effect on AA. Briefly, solvent concentration exhibits the highest effect, followed by solid-to-liquid ratio, and extraction time.

TPC and TFC are widely present plant substances and have been considered as significant contributors to AA [3], mainly due to their unique redox properties [8,14,25]. Plant TPC depends on their species/genetics and environmental conditions [26]. The F–C assay commonly used to measure the TPC; however, this assay measures the total reducing capacity of the sample, not just TPC. The TPC (as mg GAE/g) was 32.57, i.e., the highest in *C. longa* and was 2.55, i.e., the lowest in *Z. zerumbet* (Figure 3A). The ~13 times TPC difference was similar to previous studies on TPC and AA in various herbs and spices that *C. longa* had the highest TPC [23,27]. Other high TPC herbs such as *C. xanthorrhiza* [27,28], *A. purpurata* [29], *B. rotunda* [30], and *Z. cassoumounar* [31] were also reported. 

Flavonoids are a group of phenolic compounds that exhibit important biological effects and promising AA owing to their capability to scavenge reactive oxygen species effectively. The AlCl_3_ assay measures the absorbing species in the final solution, not just TFC. Among 12 selected Indonesian herbs samples, the TFC (as mg QE/g) was 279.87, i.e., the highest in *C. longa* and was 1.31, i.e., the lowest in *Z. officinale* (Figure 3B) similar to reported high curcuminoid content. Previous studies have found *lesser* TFC in *Z. officinale*, *Z. zerumbet*, and *C. heynana* [27,29,30]. The ~214 times TFC difference might be attributed to the type of flavonoids present in the herbs. The less polar ones (isoflavones, flavanones, flavones, and flavanols) could be better extracted by nonpolar solvents, whereas the more polar ones (such as glycosides and aglycones) are better extracted by alcohol or water-alcohol solvents [32]. The range of TFC is largely affected by genetic diversity, as well as biological, seasonal, and year-to-year variations [33]. 

The plant AA is caused by the presence of different antioxidant components in plant tissue. Previous studies have found that the AA of bioactive components such as TPC, tannin, anthocyanin, TFC, phenols, alkaloids, and pro-anthocyanins are mainly due to their unusual redox properties [2,8,11]. The AA was measured in an exact concentration-dependent manner based on the well-known DPPH assay [23] by reducing violet-color DPPH solution to yellow-colored product, diphenyl picryl hydrazine. The grade of color is equal to the synergetic effect by antioxidant ability and concentration of reducing components in the sample [2]. The AA (as %) was 94.85, i.e., the highest in *C. longa* and 8.45 i.e., the lowest in *Z. zerumbet* (Figure 3C). The ~11 times AA difference was close to the ~13 times TPC difference. Meanwhile, the AA (H_2_O_2_) obtained the highest value (as %), which was 64.46 in *C. longa*, and it obtained 11.27, which was the lowest in *C. manga* (Figure 3D). The different results of AA by DPPH and peroxide assay were probably due to peroxide’s weak reactivity in aqueous solutions [34]. Nevertheless, the variation pattern between these two AA results appeared similar except for *Z. zerumbet*, indicating the AA was mainly contributed by the phytochemicals or substances essential in the herbs. A notable difference was an AA (%) range, specifically, 100% for DPPH assay and 70% for H_2_O_2_ assay, presumably due to the higher sensitivity of DPPH assay.

Typical TPC contribute more to plant AA are mainly phenolic acids and flavonoids. The matrix difference among plant species from different geographical origin and variance in genetics and cultivation conditions make the correlation analysis between AA with TPC and TFC a challenging work [21,23,28,29,31,35,36]. We adopted the Pearson correlation coefficient (PCC), also referred to as Pearson’s *r*, to express the strength and direction of the linear relationship of correlation. The PCC between AA and TPC (Figure 4A) was 0.699 (*p*-value < 0.011) revealing a strong positive relationship. Similar result of PCC 0.869 (*p*-value < 0.000) between AA (H_2_O_2_) and TPC in Figure 4C, revealed a strong positive correlation. According to this result, plant AA was contributed mainly by TPC [1,3] and was highly influenced by the number and position of the hydrogen-donating hydroxyl groups. However, TPC was not the sole contributing component, and other substances cannot be ignored, especially at lower TPC (<10 mg GAE/g), which might elucidate the associated pronounced scattering extent. Another possible cause was that no sample clean up procedure was followed before TPC determination, allowing other plant substances to contribute to the TPC falsely. The PCC between AA and TFC (Figure 4B) was 0.541 (*p*-value < 0.069) revealing a moderate positive relationship, while the PCC between AA (H_2_O_2_) and TFC (Figure 4D) was 0.844 (*p*-value < 0.000), revealed a strong positive correlation. The stronger AA and TPC relationship compared to AA and TFC relationship might indicate that TFC contributes less AA than TPC, according well with previous studies that TFC plays a less substantial AA contributing role [2,3]. The PCC between TPC and TFC (Figure 4E) was 0.913 (*p*-value < 0.000) revealing an expected strong positive relationship as both classes all contribute to plant AA. Plausible contributors from other families of compounds such as anthocyanins, carotenoids, ascorbic acids, terpenoids, tannins, and tocopherols in medicinal plants [6] cannot be excluded. Confirmative analysis of these compounds warrants advanced MS analysis or using standard compounds in the future.

*C. longa*, *B. rotunda*, *A. purpurata*, *C. xanthorrhiza*, and *K. galanga* exhibiting high TPC, TFC, and AA, were further confirmed and quantified their five SPC content using HPLC-PAD. The chromatograms of the five SPC standard solutions (Figure 5A) and herbs mentioned before disclose that the highest curcumin (peak e) amount was found in *C. longa* (Figure 5C) and *C. xanthorrhiza* (Figure 5E) as reported in previous studies [23,37]. The intensive peak at 12.700 min RT in *B. Rotunda* (Figure 5F) was uncertainly assigned as antioxidant active panduratin, alpiretin, pinocembrin, or cardamonin [35,36]. The high peak at 12.191 min RT in *A. purpurata* (Figure 5D) was tentatively assigned as kaempferol, rutin, or oliconide [32]. At 12.199 min RT in *K. galangal* (Figure 5B), the peak was tentatively assigned as ethyl-*p*-methoxycinnamate [38]. Confirmative analysis of these tentatively assigned compounds warrants relevant standard compounds and elaborated MS analysis. The measured SPC content (Table 4) revealed that curcumin and quercetin were the most detected SPC. Curcumin, a flavonoid polyphenol, was present in all five herbs with the highest being 10.34 mg/g in *C. longa* and the lowest being 0.38 mg/g in *A. purpurata*. The ~10 times curcumin content difference is much larger than the ~2.5 times TPC difference. Quercetin, a flavonoid compound present in most plants [30], was present in 4 herbs (except *C. longa*) with the highest amount being 0.43 mg/g in *C. xanthorrhiza* and the lowest being 0.07 mg/g in *A. purpurata*. The ~6 times quercetin content difference was similar to the ~6.7 times TFC difference. The PCC between AA and curcumin (Figure 6A) was 0.849 (*p*-value < 0.069), and PCC between AA (H_2_O_2_) and curcumin (Figure 6C) was 0.943 (*p*-value < 0.016), revealing a strong positive relationship which indicated that AA was likely contributed mainly by curcumin. The PCC between AA and quercetin (Figure 6B) was −0.209 (*p*-value < 0.559), revealing a weak negative relationship, while the PCC between AA (H_2_O_2_) with quercetin (Figure 6D) was 0.248 (*p*-value < 0.807), revealing weak correlation. This unexpected result was presumably due to the fact that quercetin was in flavonol glycoside form such as quercetin-3-*O*-β-d-glucoside [39] which cannot be detected by the free quercetin specific HPLC-PAD method used in this study. 

The PCC between AA (H_2_O_2_) and curcumin (Figure 6C) was 0.943 (*p*-value < 0.026), revealing a strong positive relationship which indicated that AA (H_2_O_2_) was likely con-tributed mainly by curcumin. The PCC between AA (H_2_O_2_) and quercetin (Figure 6D) was 0.248 (*p*-value < 0.807), revealing a weak positive relationship. The PCC between TPC and curcumin (Figure 6F) was 0.921 (*p*-value < 0.026), revealing a strong positive linear relationship between TPC and curcumin. A strong positive linear relationship between TPC and curcumin (Figure 6E) was revealed with PCC 0.921 (*p*-value < 0.026). The practice of the mixed-method approach finds and confirms *C. longa* as the best natural antioxidant source in Indonesian indigenous *Zingiberaceae* herbs.

## 5. Conclusions

The chemical assay revealed a remarkable difference in AA, TPC and TFC in 12 Indonesian indigenous *Zingiberaceae* herbs and the highest individual measured values in *C. longa*. HPLC-PAD analysis revealed curcumin in the five top antioxidant herbs with the highest concentration in *C. longa*. Correlation analysis showed a strong positive linear relationship between AA and TPC (or curcumin) and revealed TPC (or curcumin) to be the major antioxidant compound (or component). Our integration of a mixed-method approach concludes *C. longa* as the best source of natural antioxidants in 12 Indonesian indigenous *Zingiberaceae* herbs. We argue that the mixed-method approach can be useful for antioxidant studies as it can help deal with complexity and facilitate a more profound understanding of individual results from chemical assay, instrumental analysis, and correlation to obtain more comprehensive explanations. The use of the mixed-method approach to augment the findings of solitary methods might facilitate future researchers being able to uncover deeper and hidden meanings. 

## Figures and Tables

**Table 1:** Experimental design (orthogonal array L_16_) and measured results using *C. zedoaria*.

Exp. No	Parameters			TPC (mg GAE/g)	TFC (mg QE/g)	AA (%)
	Solvent (%)	Time (min)	S-L (g/mL)			
1	50	50	0.02	4.81	8.96	12.85
2	50	60	0.04	3.87	9.27	11.35
3	50	70	0.06	2.02	3.45	9.36
4	50	80	0.08	1.51	2.59	8.86
5	60	50	0.04	3.55	7.82	11.06
6	60	60	0.02	8.25	15.83	11.06
7	60	70	0.08	1.80	4.02	7.97
8	60	80	0.06	2.30	4.60	8.96
9	70	50	0.06	2.81	14.48	10.86
10	70	60	0.08	2.79	15.25	11.45
11	70	70	0.02	7.70	25.27	15.94
12	70	80	0.04	1.95	3.63	8.86
13	80	50	0.08	3.55	14.48	14.14
14	80	60	0.06	5.14	21.75	13.84
15	80	70	0.04	4.42	21.35	13.05
16	80	80	0.02	4.45	12.76	12.65

**Table 2:** The calculated SN ratio of AA response for solvent concentration, extraction time, and solid-to-liquid ratio.

Level	Solvent (%)	Time (min)	S–L (mg/mL)
1	20.42	21.69	22.89
2	19.71	21.50	20.81
3	21.22	20.95	20.50
4	22.55	19.75	20.29
Delta	2.84	1.94	1.99
Rank	1	3	2

**Table 3:** Analytical figures of merit of HPLC-PAD method.

Compound	Retention Time (min)	LOD (µg/mL)	LOQ (µg/mL)	Repeatability (RSD%, *n* = 3)	Recovery (%)
Gallic acids	8.20 ± 0.014	0.20	0.52	<2%	98 ± 1
Naringin	10.30 ± 0.004	0.29	0.44	<5%	97 ± 4
Ferulic acids	10.41 ± 0.003	0.02	0.02	<4%	100 ± 3
Quercetin	11.24 ± 0.003	0.02	0.02	<4%	100 ± 4
Curcumin	12.02 ± 0.002	0.18	0.33	<6%	98 ± 5

**Table 4:** SPC content (mg/g) measured by HPLC-PAD.

SPC	Gallic Acid	Naringin	Ferulic Acid	Quercetin	Curcumin
*C. longa*	ND	ND	ND	ND	10.34 ± 0.02
*B. rotunda*	ND	ND	ND	0.29 ± 0.007	0.61 ± 0.06
*A. purpurata*	ND	ND	ND	0.07 ± 0.002	0.38 ± 0.022
*C. xanthorrhiza*	ND	ND	0.04 ± 0.005	0.43 ± 0.026	3.78 ± 0.553
*K. galangal*	ND	ND	ND	0.32 ± 0.02	0.99 ± 0.08

ND: not detected.

## Data Availability

Data is contained within the article.
